# Together for the Better: Improvement of a Model Based Strategy for Grapevine Downy Mildew Control by Addition of Potassium Phosphonates

**DOI:** 10.3390/plants9060710

**Published:** 2020-06-02

**Authors:** Gottfried Bleyer, Fedor Lösch, Stefan Schumacher, René Fuchs

**Affiliations:** Department of Biology, State Institute of Viticulture and Enology, Merzhauser Str. 119, 79100 Freiburg, Germany; fedor.loesch@fliegauf.com (F.L.); stefan.schumacher@wbi.bwl.de (S.S.); rene.fuchs@wbi.bwl.de (R.F.)

**Keywords:** grapevine, *Plasmopara viticola*, fungicide, copper, VitiMeteo, disease modelling, plant protection, potassium phosphonates, downy mildew, viticulture

## Abstract

Grapevine downy mildew is one of the major diseases in viticulture. To control this disease, a more effective strategy has been developed and established based on growth and model data as well as on a combination of fungicides. For this purpose, the systemic plant protection product potassium phosphonate (PP) was combined with two contact fungicides. Treatments were carried out according to the different experimental conditions after the growth of 400 cm^2^, 600 cm^2^, and 800 cm^2^ leaf area per primary shoot. PP increased the effectiveness of the preventive fungicides whenever high infection pressure was the case. The experiments also show that it is possible to extend the treatment intervals from 400 cm^2^ to 600 cm^2^ new leaf area when PP was added. However, none of the tested treatments were sufficient for the extension to intervals of 800 cm^2^. These data show that PP can be a key factor in the reduction of the application of synthetic or copper-based fungicides.

## 1. Introduction

Grapevines are grown on approximately 7.4 million hectares of land worldwide [1]. Most of this area is cultivated with *Vitis vinifera* L. which has been used for human consumption for more than 5000 years [2]. Today, more than 10,000 cultivars of *Vitis vinifera* are known, which have been developed through natural crossings, somatic variation events, and breeding, but only 2500 are used for wine production or direct consumption all over the globe [3]. Grapevine downy mildew (GDM), caused by the obligate biotrophic oomycete *Plasmopara viticola* (Berk. & Curt. *ex.* de Bary), is one of the most challenging diseases in viticulture, especially in humid climates. Under favorable weather conditions with temperatures above 13 °C and more than 95% humidity or persistent leaf wetness, *P. viticola* can cause new infections every five to seven days [4]. Since *P. viticola* and *V. vinifera* developed on different continents and were mutually exposed just 150 years ago [5], the plants were never challenged to develop any defenses against this pathogen during evolution [6]. Therefore, almost all cultivars of *V. vinifera* are highly susceptible to downy mildew, which may consequently lead to complete loss in untreated vineyards [7].

While GDM control in organic viticulture is limited to copper-based fungicides, conventional viticulture relies mainly on synthetic fungicides [8]. Governmental regulations, such as the National Action Plan on the Sustainable Use of Plant Protection Products for EU Member states, demand the reduction of pesticides under the guidelines of integrated plant protection to the minimum rational amount [9]. Valuable tools for integrated plant protection and the reduction of treatments are decision support systems (DSS), e.g., Agrometeo.ch [10], vite.net [11], or VitiMeteo [12]. Combining real time weather data, weather forecast models, and specific algorithms, the well-established DSS VitiMeteo provides predictions of the development of the most important diseases and pests in viticulture in several winegrowing countries in Europe [13]. Based on predicted infection events and the calculated development of plant growth, winegrowers are able to decide whether a treatment is necessary or not. Building on the data provided by VitiMeteo, the State Institute of Viticulture and Enology (WBI; Freiburg, Germany) developed a plant protection strategy for the safe and effective control of GDM. Treatment intervals within this strategy mainly rely on the area of new grown leaves per primary shoot [12,14]. The protection achieved with one spray treatment is maintained as long as the expansion of leaf area is below 300–400 cm^2^ new grown leaf area per primary shoot (NLA), which corresponds to two to three new leaves. In the case that no infection is predicted, the treatment interval can be prolonged until the next critical event, e.g., when rain or prolonged leaf wetness take place. If the leaf area expands above this threshold and a rather weak infection is predicted, a new spray is recommended after 80% of the pathogen incubation time has passed but as early as possible before rain. Fungicide choice should be made with regard to infection pressure, phenology, and weather forecast. In the case of strong infections, a spray treatment with a curative fungicide at the next possible date is recommended.

During this study, field trials were carried out for three consecutive years in 2014, 2015, and 2016. The aim was to further expand the treatment period from 400 cm^2^ up to 600 cm^2^ and 800 cm^2^ NLA, respectively. For this purpose, two different contact fungicides were tested in combination with potassium phosphonates (PP), which showed a strong direct effect against GDM when applied in leaf disc assays [15]. All experiments were conducted with the product Folpan^®^, a synthetic fungicide based on the phthalimide folpet and with the product Cuprozin progress^®^, a product based on copper hydroxide which is widely applied in organic viticulture. Since potassium phosphonate has no longer been allowed in organic winegrowing since 2013, organic winegrowers face increasing problems during years with high infection pressure. The results of this study show that PP can improve the effect of contact fungicides and that it could be an important option to ensure yields in organic farming in the event of a possible re-approval.

## 2. Results

**Figure 1:**
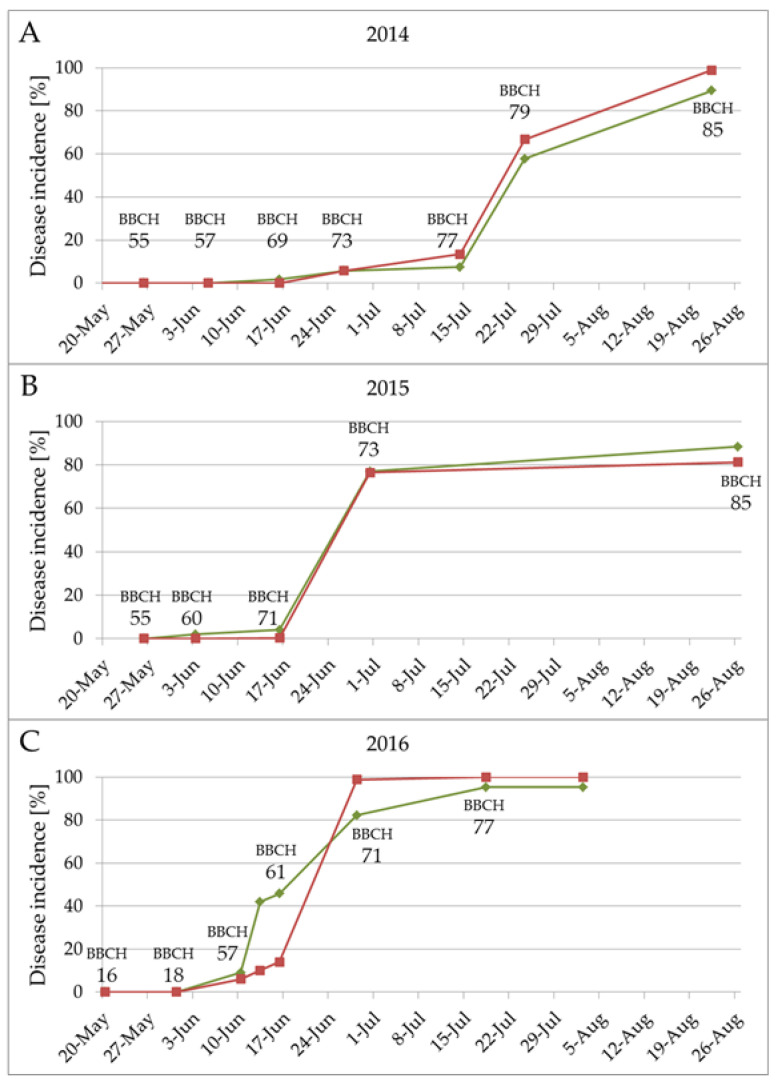
Epidemiology of *P. viticola* differed in the years 2014, 2015, and 2016. Graphs show the disease incidence of *P. viticola* on *V. vinifera* cultivar (cv.) Mueller–Thurgau in the vegetation periods 2014 (**A**), 2015 (**B**), and 2016 (**C**). Green line represents results for leaves, red lines for grapes. Observation in 2016 was stopped on August 3rd due to incidence of >95%.

From 2014 to 2016, experimental plots in a vineyard in Freiburg (Germany) were controlled against *P. viticola* with different plant protection products at time points representing the different NLA periods. The first part of the experiment was conducted to evaluate the maximum timespan between two plant protection sprayings and consequently adjust the plant protection strategy against *P. viticola*. In the second part of the experiment, PP (VeriPhos^®^) was combined with the contact fungicides folpet (Folpan^®^) or copper hydroxide (Cuprozin progress^®^) to evaluate the positive additional effect of this compound for downy mildew control. To evaluate disease pressure, the epidemiology of the pathogen was observed in an untreated control each year. Downy mildew pressure differentiated significantly between the three vegetation periods (Figure 1). Due to the relatively low rainfall at the beginning of June 2014, the disease incidence developed slowly to reach approximately 10% by 15th July (BBCH 77) and then, due to constant rain during the first weeks of July (Figure 1A; Supplemental Figure S1), rapidly increased to about 60% within the next week (BBCH 79). In 2015, the development of the disease started earlier due to the more favorable weather conditions (Figure 1B, Supplemental Figure S2). Disease incidence was less than 5% until 17th June (BBCH 71) and developed to almost 80% on 1st July (BBCH 73).

Frequent rain events from bud break (BBCH 17) almost until the closing of the grapes (BBCH 79) in 2016 (Supplemental Figure S3) resulted in this being the year with the highest infection pressure during the observed period (Figure 1C). Disease incidence was around 10% already on 10th June (BBCH 57) and developed to 70% within the following 14 days (BBCH 68).

**Figure 2:**
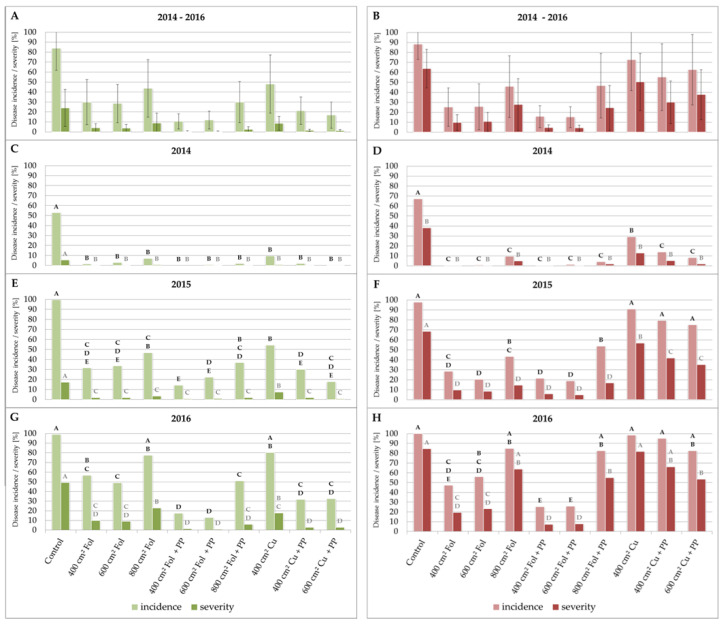
Potassium phosphonates improved the effect of contact fungicides against grapevine downy mildew (GDM). Graphs show the disease incidence and severity of *P. viticola* in leaves and berries of *V. vinifera* cv. Mueller–Thurgau after the application of different fungicides in the years 2014 (**C**,**D**), 2015 (**E**,**F**), and 2016 (**G**,**H**). Green bars show results for leaves, red bars for berries. Different letters indicate significant differences between the treatments while black letters refer to disease incidence and grey letters to disease severity (one-way ANOVA; *p* ≤ 0.05). (**A**,**B**) show average values from all three years which were subject to large variability and therefore show no significant differences between the treatments. Cu = Cuprozin progress^®^, Fol = Folpan^®^, PP = potassium phosphonates.

The increase in the efficiency of the combination of PP with a contact fungicide was analyzed with Folpan^®^ or Cuprozin progress^®^. The results show that both products clearly benefited from the addition of PP if used for GDM control. When Folpan^®^ sprayings were performed after every 400 cm^2^ NLA, the addition of PP reduced the average disease incidence in leaves from 30% (disease severity 4%) to 11% (disease severity 1%) (Figure 2A). The effect of Cuprozin progress^®^ in leaves was also greatly increased, leading to a decrease in disease incidence from 48% (disease severity 9%) to 21% (disease severity 1%). The effect on berries was not as obvious as in leaves (Figure 2B). On average, PP addition in the Folpan^®^ treatment reduced the disease incidence from 25% (10% disease severity) to 16% (4% disease severity). In the Cuprozin progress^®^ treatment, disease incidence was reduced from 73% (50% disease severity) to 63% (38% disease severity) after the addition of PP. However, due to the divergence in GDM epidemiology between the three years, significant differences between the treatments are only visible if the years are considered individually.

Since 2016 was a particularly favorable year for the development of the pathogen, the positive effects of additional PP, as well as the differences between the time points of treatments and the different products, were highly visible during this season (Figure 2G,H). The untreated control in 2016 reached over 80% disease incidence in leaves and 100% in berries already at the end of June. At the end of the experiment, the control showed a disease incidence of 99% (49% disease severity) in leaves and 100% (85% disease severity) in berries. The addition of PP to the Folpan^®^ treatments in leaves every 400 cm^2^ NLA resulted in a significant decrease in disease incidence, from 57% to an incidence of 17%. Changes in disease severity (from 10% to 1%) were clearly visible but not significant due to a rather high variability in the Folpan^®^ treatment. In the case of Cuprozin progress^®^ in leaves, the effect of PP was even clearer and improved the effect on both disease severity and incidence significantly. Treatment with Cuprozin progress^®^ alone resulted in a disease incidence of 80% (18% disease severity). The addition of PP reduced the disease incidence to 32% (3% disease severity). Considering the development of the disease in berries, Folpan^®^ treated grapes showed a disease incidence of 47% (19% disease severity) which reduced to 25% (7% disease severity) when combined with PP. The Cuprozin progress^®^ treatment in berries resulted in a disease incidence of 99% (82% disease severity) and decreased to 95% (66% disease severity). However, differences between the treatments with or without PP in berries were visible but only statistically significant in the case of Folpan treatment after 600 cm^2^.

In 2015, disease development was slower compared to 2016 (Figure 1B). At the end of the experiment, the control showed a disease incidence of 99% (17% disease severity) in leaves (Figure 2e) and 98% (68% disease severity) in berries (Figure 2F). Folpan^®^ treatment in leaves resulted in an incidence of 32% (2% disease severity) which was reduced to an incidence of 14% (1% disease severity) when PP was added. Significant differences in leaves were measured for the Cuprozin progress^®^ treatment where PP reduced the disease incidence in leaves from 54% (7% disease severity) to 30% (3% disease severity). Considering the berries, significant differences were only observed in disease severity between the Cuprozin progress^®^ (57%; incidence 91%) and the Cuprozin progress^®^ plus PP (42%; incidence 79%) treatments. Folpan^®^ treated berries showed a disease incidence of 28% (10% disease severity) which reduced to 21% (6% disease severity) when combined with PP.

Due to very unfavorable weather conditions for the development of the pathogen in 2014, disease progress was slow (Figure 1A, Supplemental Figure S1). At the end of the experiment, the control showed a disease incidence of 53% (5% disease severity) in leaves (Figure 2C) and 67% (38% disease severity) in berries (Figure 2D). Due to the rather low infection pressure, both treatments, Folpan^®^ alone and Folpan^®^ combined with PP, protected the leaves equally. In addition, Cuprozin progress^®^ alone and Cuprozin progress^®^ combined with PP showed no significant differences in leaves. Considering the berries, significant differences were only observed in disease severity between the Cuprozin progress^®^ (13%; incidence 29%) and the Cuprozin progress^®^ plus PP treatments (5%; incidence 14%). Folpan^®^ treated berries showed no symptoms due to the low infection pressure.

To estimate the minimal interval of sprayings in correspondence to new grown leaf area, treatments were performed between BBCH 14 and BBCH 73 (Table 1 and Table 2). During this period, leaf area expanded by 2400 cm^2^ per main shoot. Treatments were performed after 400 (2–3 leaves), 600 (4 leaves), and 800 cm^2^ (5–6 leaves) of new grown leaf area. The results of the field trials indicate that plots treated five times (every 600 cm^2^) showed similar disease incidence and severity as plots treated seven times (every 400 cm^2^). However, four treatments (every 800 cm^2^) were not sufficient for an effective control (Figure 2).

## 3. Discussion

Downy mildew control is a challenging task for winegrowers in humid climates. Disease models, more and more precise weather forecast data, and consequently increasingly reliable decision support systems have become valuable tools for an effective plant protection strategy. However, in order to provide reliable predictions, DSS depend on accurate and reliable data provided by local and fully maintained weather stations. One well established decision support system in viticulture which combines several models for diseases, pests, and phenology is VitiMeteo (VM) [13]. VitiMeteo was introduced in 2002 and has now been evaluated in vineyards throughout Baden-Wuerttemberg (Germany) for almost two decades. Since both the weather stations and the platform are publicly funded, VitiMeteo provides winemakers with a freely accessible, free and independent tool. Given that the purpose of VitiMeteo is not to relieve the winemakers from making a self-determined decision, they must decide for themselves on the extent and necessity of treatment and are therefore continuously trained in the use of the platform. In the case of downy mildew, the model VM Plasmopara calculates upcoming infection events and the time span of incubation but also phenology and the development of leaf area [12]. For the most effective implementation of this system in practice, a plant protection strategy based on the provided data was developed and improved during the last few decades [14]. As mentioned above, spray intervals in this strategy are determined by the area of new grown leaves per primary shoot (NLA). The aim of this work was to further optimize this strategy in order to achieve a further reduction in the number of fungicide treatments.

Field experiments in this study were performed during the years 2014, 2015, and 2016. While the original plant protection strategy recommended treatment after 400 cm^2^ under strong infection pressure, the results here show that an extension to 600 cm^2^ is possible without loss of efficacy. Due to particularly favorable weather conditions in 2016, the GDM epidemic developed very early and quickly, resulting in a season with an unusually high infection pressure. The year 2016 was therefore particularly well suited to uncovering the differences between the various treatments.

As expected, the addition of PP improved the effect of all the treatments against GDM. Considering disease incidence and severity in leaves, treatment with Folpan^®^ plus PP after 800 cm^2^ NLA was as good as treatment with Folpan^®^ after 400 cm^2^ NLA. However, this effect was not observable in berries where disease incidence and severity in both treatments after 800 cm^2^ NLA was significantly higher than in the treatments after 400 cm^2^ and 600 cm^2^. Under the strong infection pressure of 2016, the combinations of Folpan^®^ and PP resulted in a disease incidence beneath 20% with a severity of 1% and therefore almost healthy leaves. The effect on berries was also remarkable, showing a disease incidence under 25% with a severity clearly under 10%. In the case of Cuprozin progress^®^, the addition of PP also significantly improved the effect on leaves. Remarkably, in berries Cuprozin progress^®^ was not able to perform significantly better than the control, while the combination with PP clearly reduced the disease in the vineyard. Differences between the spray treatments performed after 400 cm^2^ and after 600 cm^2^ NLA were not visible. Although growth-based decisions for plant protection treatments allowed very effective planning of spray intervals during the season, this strategy should only be implemented over the main growth phase between BBCH 13–16 (three to six leaves unfolded) and BBCH 73 (grain size of the berries). After BBCH 73, leaf area expands rather slowly and treatments should be scheduled according to the number of new grown leaves (e.g., two to three new leaves) rather than by leaf area (data not shown).

Even though PP was able to improve the effect of both fungicides, its overall performance in berries was lower than in leaves. Since PP is distributed in the plant via phloem and xylem, one would assume a systemic effect also on berries [16,17]. Although PP based plant protection products have been available since the 1970s, their mode of action is not exactly clear [17]. Several studies claim that PP may induce a defense reaction in plants, e.g., the induction of glucanase in grapevines [18,19,20,21]. Since no studies on the possible differences in induced resistance between grapes and leaves have been conducted, one can only speculate here. Several defense reactions of the plant were observed and measured after PP treatment, for example, papilla formation, lignification, ethylene biosynthesis, phenylalanine ammonia lyase activation, or accumulation of phytoalexins [17], but have never been analyzed in berries in detail. However, grapevines treated with the phosphonate fosetyl-aluminum showed only a very weak induction of defense-related genes [22].

Besides the effects of PP linked to an induction of resistance in the plant, the direct effects of PP on pathogens, like several *Phytophthora* species, have been shown [17,23]. The curative effect against GDM has been known for almost 30 years [24,25]. This effect may be concentration dependent. Since potassium phosphonates are mainly absorbed by the leaves, their concentrations there may be higher than in berries, leading to a better effect during GDM control.

Even though PP works more effectively in leaves, its residues are detectable in fruits or their end products like wine [26,27]. This is also why PP was not authorized as a plant protection product in organic farming [28]. As mentioned above, organic viticulture depends mainly on copper-based fungicides for GDM control. Particularly difficult years, such as 2016, therefore pose major problems for organic winegrowers in Europe, especially since the amount of copper was reduced to a maximum application rate of 28 kg/ha over a period of 7 years (i.e., on average 4 kg/ha/year).

The results of this study show that the addition of PP can significantly reduce fungicide dosage in GDM control. PP has a medium to high persistence in soil and the risk to soil-dwelling organisms was determined as low. This is also true for the risk to aquatic organisms [29]. PP can increase economic viability for organic winegrowers and be a promising addition to copper-based fungicides in difficult years. With regard to the direct negative effects of copper on soil microorganisms and insects or the possible indirect negative effects on the health of animals like sheep in double-used vineyards [30,31], PP may be a promising component for copper reduction in plant protection, especially since the acute toxicity of phosphoric acid is low and no safety concerns regarding its dietary intake through grape or wine have been detected [29].

## 4. Materials and Methods

Field trials with ten different experimental conditions were conducted in three consecutive years from 2014 to 2016 (Table 1 and Table 2). The execution and evaluation of the experiments were carried out after the published standards of the European and Mediterranean Plant Protection Organisation (EPPO). Each experimental condition was tested on 64 vines in four repetitions (4 × 16 vines). All trials were performed on the susceptible *Vitis vinifera* cultivar Mueller–Thurgau in an experimental vineyard in Freiburg (47°58′40.7″ N 7°50′01.8″ E). The vineyard was planted in 2011 with 5000 plants/ha (row spacing 2 m, plant spacing 1 m) in an Espalier-type system on loam soil. Artificial infections were made to ensure a uniform and high infection pressure in the field (29 April 2014; 7 and 20 May 2015; 11 May 2016). Therefore, one single leaf from every fourth plant was infected by spray infection with a sporangia solution with a *P. viticola* isolate from WBI Freiburg (Germany). To ensure the viability of the isolate, it was refreshed with collected sporangia every year. The sporangia solution was produced by the rinsing of infected leaves with desalted water and was adjusted to a concentration of 25,000 sporangia/mL. Infection was achieved with a commercially available pump-sprayer on a spot that was 3 cm in diameter on the lower side of the leaf. For incubation, the leaf was wrapped in a plastic bag over night. The first treatment was carried out just before the end of the incubation period (Supplemental Table S1). The following treatments were carried out according to the growth model of H.-R. Schulz [32] of VitiMeteo, depending on the variant after 400 cm^2^, 600 cm^2^, or 800 cm^2^ new grown leaf area per main shoot. To achieve high infection pressure, artificial irrigation of the vineyard with overhead sprinklers was performed in the absence of rain one or two days before the planned treatment with 29 mm water overnight as well as in times with little rain to boost the infections (Supplemental Table S2).

Spray treatments were performed with the commercially available fungicides VeriPhos^®^ (755 g/L potassium phosphonates; Adama GmbH, Germany), Folpan^®^ 80 WDG (800 g/kg Folpet; Adama GmbH, Germany), and Cuprozin^®^ progress (383.8 g/L copper hydroxide; Spiess-Urania Chemicals GmbH, Germany). Treatments were performed with a tunnel-sprayer (Schachtner, Ludwigsburg, Germany) with TeeJet XR80015VS nozzles (TeeJet Technologies, Schorndorf, Germany). The application rate was adjusted according to the phenological state from the 1× basic application rate at BBCH 16 (1× concentrated in 400 L/ha) to 3.5× basic application rate at BBCH 73 (1.75× concentrated in 800 L/ha). Valuation was carried out after the European and Mediterranean Plant Protection Organization (EPPO) standards in four repetitions per treatment in a randomized vineyard. The severity of the disease was estimated for every experimental condition by visually determining the percentage of symptomatic leaf/grape surface area on 4 × 100 leaves or grapes. The disease incidence was calculated by dividing symptomatic leaves or grapes by the total number of leaves or grapes examined. Statistical analysis for significance was performed by one-way analysis of variance (ANOVA; *p* ≤ 0.05).

## Figures and Tables

**Table 1:** Basic parameters of the field trials between 2014 and 2016.

Date of First Treatment (BBCH)	Date of Last Treatment (BBCH)	Number of Days from First to Last Treatment	New Grown Leaf Area [cm^2^]	Natural Precipitation [mm]	Number of Nights with Irrigation	Artificial Irrigation (Sprinkling [mm])	∑ Precipitation [mm]
6 May 2014 (15)	23 June 2014 (73)	48	0–2400	40	10	305	345
12 May 2015 (16)	29 June 2015 (73)	48	0–2400	167	8	217	384
17 May 2016 (14)	29 June 2016 (71)	43	0–2400	235	6	170	405

**Table 2:** Treatment schedule of the different experimental conditions according to new grown leaf area (NLA).

**Var. No**.	No. of treatment every 400 cm^2^ NLA	1	2		3	4	5		6	7
	No. of treatment every 600 cm^2^ NLA	1		2		3		4		5
	No. of treatment every 800 cm^2^ NLA	1			2		3			4
	BBCH-code	14–16								71–73
	NLA [cm^2^]	0	400	600	800	1200	1600	1800	2000	2400
**1**	Untreated control	-	-	-	-	-	-	-	-	Folpan^®^
**2**	400 cm^2^ Folpan^®^	X	X	-	X	X	X	-	X	X
**3**	400 cm^2^ Folpan^®^ + PP	X	X	-	X	X	X	-	X	X
**4**	400 cm^2^ Cuprozin progress^®^	X	X	-	X	X	X	-	X	X
**5**	400 cm^2^ Cuprozin progress^®^ + PP	X	X	-	X	X	X	-	X	X
**6**	600 cm^2^ Folpan^®^	X	-	X	-	X	-	X	-	X
**7**	600 cm^2^ Folpan^®^ + PP	X	-	X	-	X	-	X	-	X
**8**	600 cm^2^ Cuprozin progress^®^ + PP	X	-	X	-	X	-	X	-	X
**9**	800 cm^2^ Folpan^®^	X	-	-	X	-	X	-	-	X
**10**	800 cm^2^ Folpan^®^ + PP	X	-	-	X	-	X	-	-	X

## Supplementary Materials

The following supplemental figures and tables are available online at https://www.mdpi.com/2223-7747/9/6/710/s1, Figure S1: Weather data and experimental design 2014, Figure S2: Weather data and experimental design 2015, Figure S3: Weather data and experimental design 2016, Table S1: Date of treatments, Table S2: Artificial irrigation.
