# Correction: Bleyer et al. Together for the Better: Improvement of a Model Based Strategy for Grapevine Downy Mildew Control by Addition of Potassium Phosphonates. *Plants* 2020, *9*, 710

**DOI:** 10.3390/plants13101369

**Published:** 2024-05-15

**Authors:** Gottfried Bleyer, Fedor Lösch, Stefan Schumacher, René Fuchs

**Affiliations:** Department of Biology, State Institute of Viticulture and Enology, Merzhauser Str. 119, 79100 Freiburg, Germany; fedor.loesch@fliegauf.com (F.L.); stefan.schumacher@wbi.bwl.de (S.S.); rene.fuchs@wbi.bwl.de (R.F.)

**Error in Figure**

Figure 2 was incorrect in the original publication [1]. Bars indicating disease incidence and disease severity of the treatments “400 cm^2^ Cu + PP” and “600 cm^2^ Cu + PP” were interchanged in Figure 2C–F. Consequently, the average values presented in Figure 2A,B for treatments “400 cm^2^ Cu + PP” and “600 cm^2^ Cu + PP” were incorrect. The corrected Figure 2 is shown below.

**Changes in the text due to correction of Figure 2**

Due to above mentioned mistake in Figure 2A–F, several sentences in the results section of the publication had to be revised. 

On page 3, the sentence “The effect of Cuprozin progress^®^ in leaves was also greatly increased, leading to a decrease in disease incidence from 48% (disease severity 9%) to 21% (disease severity 1%).” was changed to “The effect of Cuprozin progress^®^ in leaves was also greatly increased, leading to a decrease in disease incidence from 48% (disease severity 9%) to 17% (disease severity 1%).”. Furthermore, the sentence “In the Cuprozin progress^®^ treatment, disease incidence was reduced from 73% (50% disease severity) to 63% (38% disease severity) after the addition of PP.” was changed to “In the Cuprozin progress^®^ treatment, disease incidence was reduced from 73% (50% disease severity) to 59% (34% disease severity) after the addition of PP.”.

On page 5, “Significant differences in leaves were measured for the Cuprozin progress^®^ treatment where PP reduced the disease incidence in leaves from 54% (7% disease severity) to 30% (3% disease severity). Considering the berries, significant differences were only observed in disease severity between the Cuprozin progress^®^ (57%; incidence 91%) and the Cuprozin progress^®^ plus PP (42%; incidence 79%) treatments.” was changed to “Significant differences in leaves were measured for the Cuprozin progress^®^ treatment where PP reduced the disease incidence in leaves from 54% (7% disease severity) to 18% (1% disease severity). Considering the berries, significant differences were only observed in disease severity between the Cuprozin progress^®^ (57%; incidence 91%) and the Cuprozin progress^®^ plus PP (35%; incidence 75%) treatments.”. Furthermore, the sentence “Considering the berries, significant differences were only observed in disease severity between the Cuprozin progress^®^ (13%; incidence 29%) and the Cuprozin progress^®^ plus PP treatments (5%; incidence 14%).” was revised to “Considering the berries, significant differences were only observed in disease severity between the Cuprozin progress^®^ (13%; incidence 29%) and the Cuprozin progress^®^ plus PP treatments (2%; incidence 8%).”.

The authors apologize for any inconvenience caused by the mistakes listed and state that the scientific conclusions are unaffected. This correction was approved by the Academic Editor. The original publication has also been updated.

## Figures and Tables

**Figure 2:**
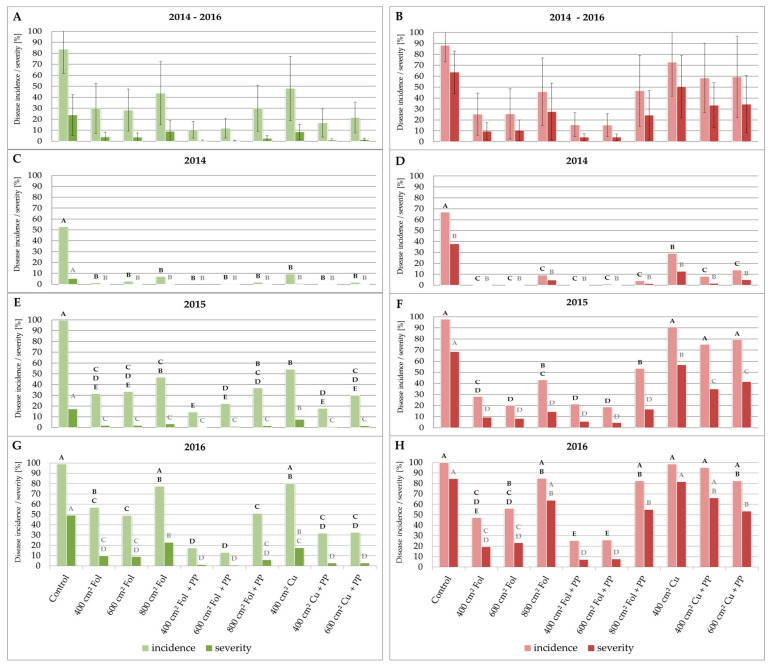
Potassium phosphonates improved the effect of contact fungicides against grapevine downy mildew (GDM). Graphs show the disease incidence and severity of *P. viticola* in leaves and berries of *V. vinifera* cv. Mueller–Thurgau after the application of different fungicides in the years 2014 (**C**,**D**), 2015 (**E**,**F**), and 2016 (**G**,**H**). Green bars show results for leaves, red bars for berries. Different letters indicate significant differences between the treatments while black letters refer to disease incidence and grey letters to disease severity (one-way ANOVA; *p* ≤ 0.05). (**A**,**B**) show average values from all three years which were subject to large variability and therefore show no significant differences between the treatments. Cu = Cuprozin progress^®^, Fol = Folpan^®^, PP = potassium phosphonates.
