# Supplementary material for: Together for the Better: Improvement of a Model Based Strategy for Grapevine Downy Mildew Control by Addition of Potassium Phosphonates
Source: Plants (Basel). 2020 Jun 2;9(6):710. doi: 10.3390/plants9060710 (PMC7355483; doi:10.3390/plants9060710)
Supplement: Supplementary file 1 [file plants-09-00710-s001.pdf]

plants

Supplementary Materials

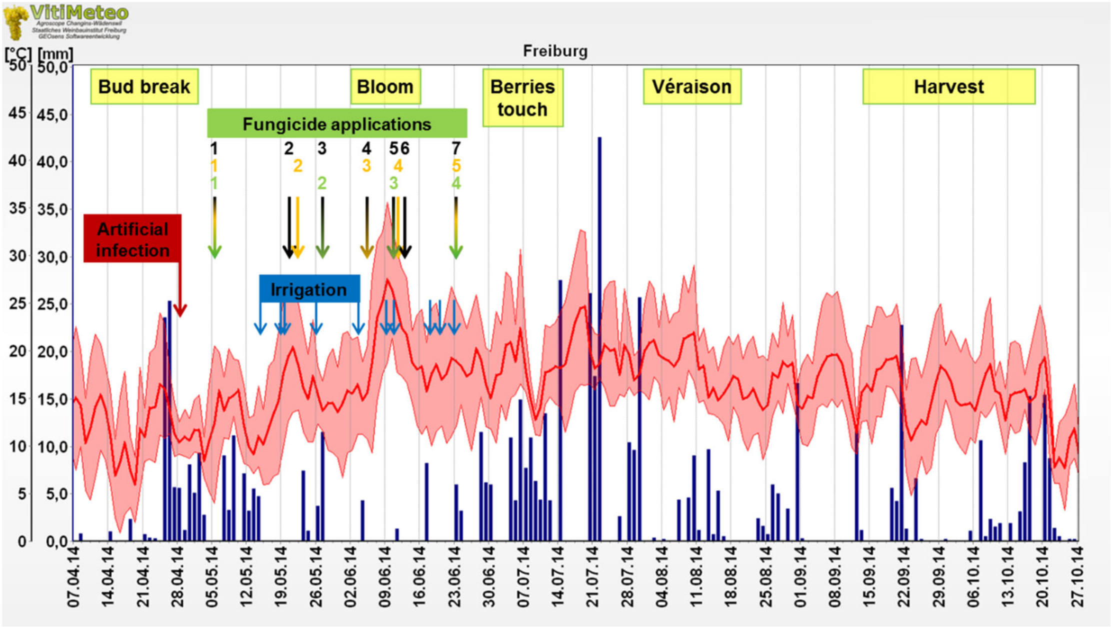

Figure S1. Weather data and experimental design 2014.

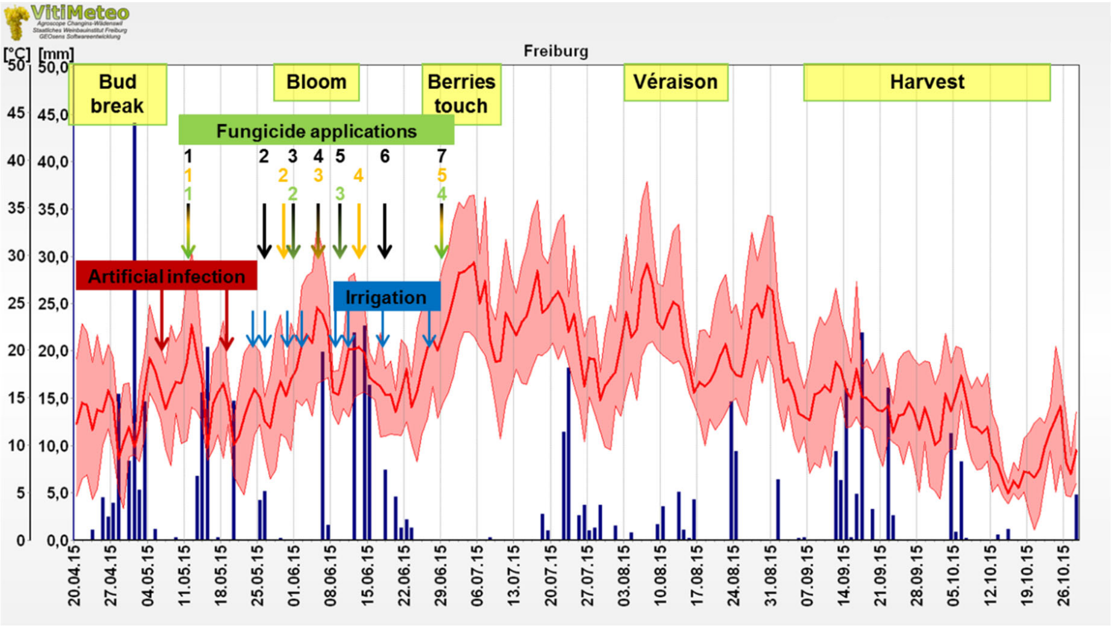

Figure S2. Weather data and experimental design 2015.

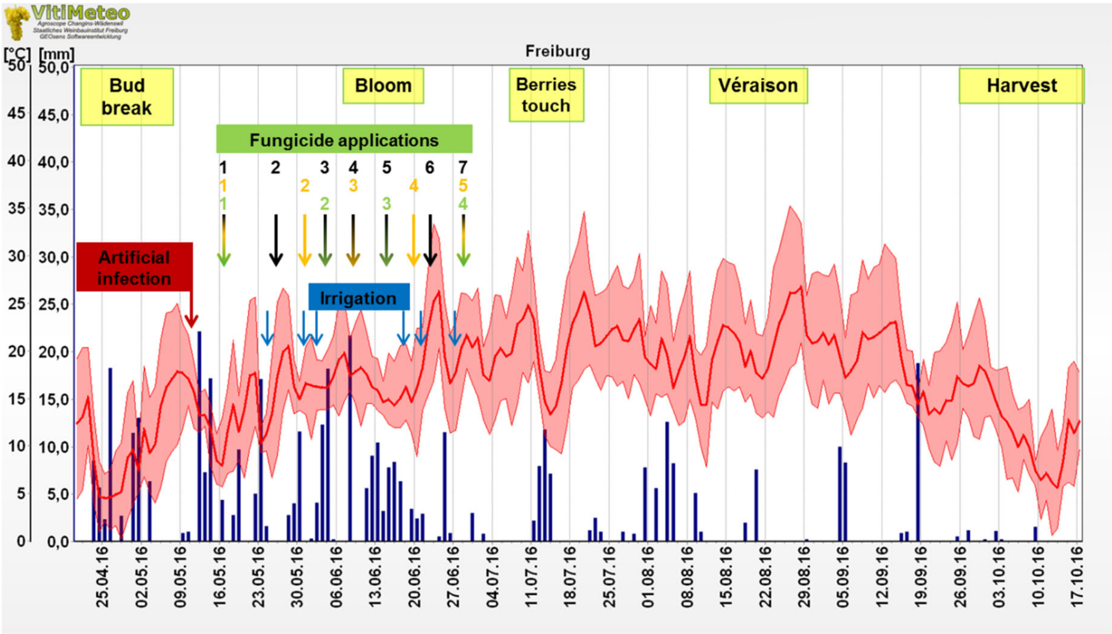

Figure S3. Weather data and experimental design 2016.

plants

MDPI

Table S1. Date of treatments.

Year	NGA [cm²]	Artificial Infection	1st Treatment	2nd Treatment	3rd Treatment	4th Treatment	5th Treatment	6th Treatment	7th Treatment	Final Folpan Treatment
	400			May 21st	May 28th	June 5th	June 10th	June 13th	June 23rd	
2014	600	April 29th	May 6th	May 23rd	June 5th	June 11th	June 23rd			June 27th
	800			May 28th	June 10th	June 23rd				
	400			May 26th	June 1st	June 5th	June 10th	June 18th	June 29th	
2015	600	May 7th + May 20th	May 12th	May 29th	June 5th	June 13th	June 29th			July 8th
	800			June 1st	June 10th	June 29th				
	400			May 27th	June 4st	June 10th	June 15th	June 23rd	June 29th	
2016	600	May 11th	May 17th	June 1st	June 10th	June 20th	June 29th			July 15th
	800			June 4th	June 15th	June 29th				

plants

MDPI

Table S2. Artificial irrigation.

No.	2014	2015	2016
1	May 15th	May 24th	May 25th
2	May 19th	May 27th	May 31st
3	May 20th	May 30th	June 2nd
4	May 26th	June 3rd	June 18th
5	June 4th	June 9th	June 21st
6	June 9th	June 11th	June 27th
7	June 11th	June 18th	
8	June 18th	June 27th	
9	June 20th		
10	June 23rd		
